# Quality of life and its associated factors among chronic disease patients in Assir, Saudi Arabia: a cross-sectional study

**DOI:** 10.7717/peerj.21592

**Published:** 2026-07-28

**Authors:** Yousef I. Zahrani, Bayapa Reddy Narapureddy

**Affiliations:** Department of Public Health, College of Applied Medical Sciences, King Khalid University, Abha, Asir, Saudi Arabia

**Keywords:** Chronic diseases, Quality of life, WHOQOL-BREF, Saudi Arabia, Health-related quality of life

## Abstract

**Background:**

Chronic diseases considerably impact physical capacity, psychological agony, and social disengagement, leading to poor health-related quality of life (HRQoL). This study aimed to comprehensively evaluate HRQoL and determine the key sociodemographic predictors associated with lower HRQoL among adults with chronic diseases in the Assir region of Saudi Arabia. Despite the rising burden of chronic diseases in Saudi Arabia, data on health-related quality of life among rural populations using a validated multidomain instrument remain sparse, and predictors specific to the Assir region are yet to be characterized.

**Methodology:**

A cross-sectional analytical study was conducted from 25 October to 23 November 2024 across five primary health-care centers (PHCCs) located in Abha, Khamis Mushait, Mahayil, Sarat Abidah, and Rijal Almaa. A total of 430 adults diagnosed with one or more chronic diseases were recruited using a quota-based random sampling approach. Data were collected using the validated World Health Organization Quality of Life-BREF (WHOQOL-BREF) questionnaire, which evaluates physical, psychological, social, and environmental domains. Binary logistic regression identified factors associated withpoor HRQoL (domain score ≤ 13), while Receiver Operating Characteristic (ROC) curve analysis assessed domain-wise discriminative accuracy.

**Results:**

The multivariable logistic regression revealed that male participants had significantly greater odds of good HRQoL compared with female (aOR = 2.46; 95% CI [1.64–3.68]; *p* < 0.001). Additionally, those who were employed had significantly greater odds of good HRQoL than those who were not working (aOR = 1.57; 95% CI [1.31–1.89]; *p* < 0.001). The analysis also found participants who were living with a spouse (aOR = 1.85; 95% CI [1.21–2.83]; *p* = 0.004), had higher education (aOR = 2.39; 95% CI [1.53–3.74]; *p* < 0.001), and had a single disease were significantly more likely to report good HRQoL (aOR = 45.99; 95 % CI [10.04–210.71]; *p* < 0.001).

**Conclusion:**

Male gender, employment status, higher level of education, living with spouse and having one chronic disease were independently related to better HRQoL, while female gender, unemployment, lower educational level, living without spouse and multimorbidity were associated with lower HRQoL.

## Introduction

Chronic diseases such as hypertension, diabetes, cardiovascular disorders, cancer, kidney disease, and chronic respiratory illnesses remain the leading causes of global morbidity and mortality (Hacker, 2024). According to the World Health Organization (WHO) (2023), approximately 41 million deaths (74%) each year are due to non-communicable diseases (NCDs), and 77% occur in low- and middle-income countries, including Saudi Arabia. Rapid urbanization, sedentary lifestyle, and population aging have contributed to the escalating chronic-disease burden in the Kingdom (Alhejely et al., 2023; Alzarea et al., 2024; Alanazi, 2020).

Health-related quality of life (HRQoL) has emerged as an essential indicator of chronic-disease management and patient-centered care (Alzarea et al., 2024; Alanazi, 2020). WHO defines HRQoL as “an individual’s perception of their position in life in the context of the culture and value systems in which they live and in relation to their goals and expectations” (The WHOQOL Group, 1998). Chronic illness affects physical, mental, and social functioning; hence, HRQoL evaluation provides a holistic measure beyond biomedical outcomes (Akyirem et al., 2022; Polis & Fernandez, 2015; Vahedi, 2010)

To measure HRQoL comprehensively, the World Health Organization Quality of Life-BREF (WHOQOL-BREF) instrument is widely utilized. It comprises 26 items grouped into four domains: physical health, psychological well-being, social relationships, and environmental factors. The Arabic version has been culturally adapted and validated among Saudi Arabian populations, demonstrating robust reliability and construct validity (Almarabheh et al., 2023). Its multidimensional nature enables clinicians and researchers to identify determinants that adversely affect patient adaptation and coping (Bratu et al., 2023; Samal et al., 2021; Anawade, Sharma & Gahane, 2024).

The Assir region, located in the southwestern highlands of Saudi Arabia, is notable for its mountainous terrain, dispersed rural settlements, and distinctive sociocultural traditions. While the region has made strides in expanding primary-care infrastructure, limited accessibility, socioeconomic disparities, and traditional health-seeking behaviors remain barriers to equitable chronic-disease management. The consequent rise in multimorbidity within rural Assir warrants a detailed assessment of HRQoL among its affected residents.

Previous Saudi studies have examined HRQoL among patients with chronic diseases, yet most were conducted in urban populations and often relied on single-domain or disease-specific instruments rather than validated multidomain measures such as WHOQOL-BREF (Ijoma et al., 2019; Qadire et al., 2023; Guallar-Castillón et al., 2005; Farzianpour et al., 2015; Ausín, Zamorano & Muñoz, 2020). Consequently, data on rural Saudi populations, particularly regarding the social and environmental dimensions of HRQoL, are sparse. Understanding these gaps is critical to inform interventions aligned with Saudi Vision 2030’s Health Sector Transformation Program (2023–2025), which prioritizes equitable, person-centered care and wellness promotion.

## Objectives

1. Assess the HRQoL of adults with chronic diseases in the Assir region using the WHOQOL BREF.
2. Evaluate the differential impact of chronic diseases across the four HRQoL domains: physical, psychological, social, and environmental.
3. Identify sociodemographic factors associated with poor HRQoL, including gender, education, employment, and multimorbidity.

We hypothesized that multimorbidity, unemployment, and female gender would independently predict lower HRQoL scores across all domains. By addressing these objectives, the study seeks to generate region-specific evidence that can guide targeted interventions for chronic-disease management and policy planning in rural Saudi Arabia.

## Methodology

**Study design**: This study employed a cross-sectional analytical design to assess the health-related Quality of Life (HRQoL) among adults diagnosed with chronic diseases in the Assir region of Saudi Arabia.

The study adhered to the principles of the STROBE (Strengthening the Reporting of Observational Studies in Epidemiology) guidelines.

**Study setting and period**: The study was conducted between 25 October and 23 November 2024 across five major Primary Health Care Centers (PHCCs) in the Assir region, namely Abha, Khamis Mushait, Mahayil, Sarat Abidah, and Rijal Almaa. These centers were selected because they serve both urban and rural populations, ensuring geographical and sociodemographic diversity. Abha, being the regional capital, provided representation from semi-urban sectors, while Mahayil, Sarat Abidah, and Rijal Almaa represented remote mountainous rural communities. This geographic selection ensured inclusion of patients with variable access to healthcare, educational levels, and lifestyle profiles. The cross-sectional approach was selected because it allows evaluation of multiple HRQoL domains and their associations with sociodemographic variables within a defined time frame, providing a snapshot of health outcomes and predictors.

Study population and eligibility criteria: The target population included adult patients aged 20 to 85 years diagnosed with at least one chronic disease and attending any of the selected PHCCs during the data collection period. Eligible participants were required to be literate in either Arabic or English and capable of completing the WHOQOL-BREF questionnaire independently or with assistance.

Inclusion criteria: Adults (≥20 years) with at least one physician-diagnosed chronic disease (*e.g.*, diabetes, hypertension, cardiovascular disease, chronic respiratory disease, hepatitis, stroke, or chronic kidney disease), Regular follow-up at any of the five PHCCs during the study period, and ability and willingness to provide informed consent. Exclusion Criteria: Individuals younger than 20 or older than 85 years, pregnant women due to temporary physiological and emotional changes affecting HRQoL, patients with acute medical conditions, dementia, or severe cognitive impairment that precluded informed participation, and Individuals with uncontrolled psychiatric illnesses were also excluded to minimize confounding from mental disorders unrelated to chronic physical illness.

**Sample size and sampling technique:** Sample size was calculated based on the following formula used to estimate a population mean: n = (Z × SD/d)2. A Z value of 1.96 was used for a confidence interval of 95%, SD was taken from previously published WHOQOL-BREF domain scores among a comparable Saudi population [14,15], and set at 14.4. The margin of error (d) was set at 1.36 or around 10% of the mean domain score. Calculation: n = (1.96 × 14.4/1.36)2 = (28.22/1.36)2 = 20.752 = 431. The final sample size was rounded down to 430 participants to allow for a statistical power ≥80% to detect significant differences between subgroups of Quality of Life (QoL) domain scores. Given that the primary outcome was a continuous HRQoL domain score rather than a binary proportion, the sample size formula for mean estimation was applied. The SD value of 14.4 was drawn from comparable Saudi HRQoL studies (Ijoma et al., 2019; Qadire et al., 2023). The final target sample was rounded off to 430 participants, providing adequate power (80%) to detect meaningful differences in QoL scores between single- and multiple-morbidity groups.

Patients were recruited through multi-stage sampling technique. Using quota sampling technique for stage one, participants were proportionally allocated to five PHCCs (about 85 participants per center, total = 430) to ensure representation from the rural and semi-urban areas socio-demographically. Sampling frame at each center was based on patients’ register of those who attended follow-up clinics and were registered with a chronic disease diagnosis in the PHCC electronic medical records system. For stage two sampling, systematic random sampling method was used in each PHCC: starting with an arbitrarily selected patient, every third eligible patient attending the PHCC on data-collection days was invited to participate in the study. Combining quota sampling with systematic random sampling within each quota stratum helped achieve representativeness and reduce opportunity-based selection bias. Participants who refused to take part in the study or were not present at their appointment were substituted with the next patient on the list.

### Data collection procedure

Trained research assistants conducted data collection under the supervision of the principal investigator. Participants were approached in the waiting areas of PHCCs and briefed about the study. Data were collected either through face-to-face interviews or *via* an online secured form for those preferring digital participation. Verbal or digital informed consent was documented in all cases. For participants with limited literacy, trained assistants read questions verbatim without interpretation or prompting, ensuring standardization and minimizing interviewer bias.

The average time to complete the questionnaire was approximately 20 min.

### Data collection tool

The study utilized the World Health Organization Quality of Life-BREF (WHOQOL-BREF) instrument. This 26-item questionnaire assesses four major domains of QoL:

1. Physical Health (activities of daily living, fatigue, dependence on medical treatment, pain, mobility, sleep, work capacity)
2. Psychological Health (positive and negative feelings, self-esteem, body image, personal beliefs)
3. Social Relationships (personal relationships, social support, sexual activity)
4. Environment (financial resources, physical safety, healthcare access, home environment, leisure opportunities, transport)

The instrument also includes two global items on overall quality of life and general health. The Arabic version, validated among Saudi populations, was used for most participants (*n* = 395), while 35 participants opted for the English version. Both versions demonstrated excellent psychometric reliability and conceptual equivalence.

Internal consistency reliability was evaluated using Cronbach’s alpha coefficients, which were 0.89 (physical), 0.82 (psychological), 0.78 (social), and 0.80 (environmental domains), indicating excellent internal reliability. The construct validity of the Arabic WHOQOL-BREF version has been established in multiple studies conducted in the Gulf region (Ausín, Zamorano & Muñoz, 2020). A pilot test involving 30 participants was conducted before the main data collection to ensure clarity and cultural appropriateness of the instrument. No major modifications were required.

### Scoring and categorization

Each WHOQOL-BREF domain score was calculated using the standard formula:

• **Physical Health (Domain 1):** 4 ×[(6−Q3)+(6−Q4)+Q10+Q15+Q16+Q17+Q18]/7
• **Psychological Health (Domain 2):** 4 ×[Q5+Q6+Q7+Q11+Q19+(6−Q26)]/6
• **Social Relationships (Domain 3):** 4 ×[Q20+Q21+Q22]/34 \times [Q20 + Q21 + Q22] / 34 ×[Q20+Q21+Q22]/3
• **Environment (Domain 4):** 4 ×[Q8+Q9+Q12+Q13+Q14+Q23+Q24+Q25]/8

All raw scores were transformed into a 0–100 scale following WHO guidelines, where higher scores represent better quality of life. Domain scores in the present study were kept on the raw transformed scale (range 4–20) instead of the converted 0–100 scale, to align with how scores were calculated in the Saudi and regional studies used for validation. Similarly, a cut-off score of 13 on the raw scale was used to dichotomize HRQoL as good (>13) *vs.* poor (≤13) which had been validated and utilized in previous studies among chronic-disease populations in Saudi Arabia and other Gulf countries (Elshamy et al., 2023; Alavinia & Burdorf, 2008). This cut-point represented the mid-point sensitivity for identifying clinically meaningful impairment in this population and was also confirmed by ROC curve analysis conducted in this study (total AUC = 0.868) as providing good discriminatory validity.

**Ethical approval and considerations**: The study protocol was reviewed and approved by the King Khalid University Research Ethics Committee (Approval No.: ECM#2024-2802).

All participants provided informed consent, either written or digital, after receiving an explanation of the study objectives, procedures, and confidentiality assurances. The study was conducted in accordance with the Declaration of Helsinki. Participants were informed that their participation was voluntary and that they could withdraw at any time without consequences for their medical care. Anonymity was ensured by coding each questionnaire, and personal identifiers were removed prior to data entry. For participants who experienced distress while completing the questionnaire, mental health helpline contact information and on-site counseling options were provided.

### Statistical analysis

Data were coded and entered into SPSS version 27 (IBM Corp., Armonk, NY, USA) using a double-entry method to minimize transcription errors. Descriptive statistics (frequency, percentage, mean, and standard deviation) were used to summarize participant characteristics and domain scores. The Chi-square (*χ*^2^) test was applied to examine associations between categorical variables and HRQoL categories. One-way ANOVA compared mean domain scores across demographic subgroups. At a 95% Confidence Interval, and a *p*-value <0.05 was considered statistically significant. Multivariate logistic regression analyses were conducted to determine factors independently associated with poor HRQoL (domain score ≤ 13), presenting estimates as adjusted odds ratios (aOR) with 95% confidence intervals (CI). The Hosmer–Lemeshow goodness-of-fit test was used to assess how well the data fitted the final model. Multicollinearity was tested using variance inflation factor (VIF); values of VIF < 2.5 indicated no evidence of multicollinearity. Receiver Operating Characteristic (ROC) curve analyses were then carried out to determine how accurately each WHOQOL-BREF domain discriminated HRQoL.

## Results

### Sociodemographic characteristics of participants

A total of 430 participants with one or more chronic diseases were included in the study. The mean age of participants was 58.13 (10.92) years, ranging from 22 to 84 years. Of the total participants, 257 (59.7%) were male, and 173 (40.3%) were female. The majority (77.4%) were married, and approximately three-fourths (74.6%) were unemployed or retired. Educational status varied across age groups, with the highest proportion of individuals holding a bachelor’s degree (61.2%), followed by college/diploma holders (21.2%) and high school graduates (17.6%). Most participants (79.7%) had a single chronic disease, while 20.3% had two or more chronic diseases.

The proportion of males was highest in the extreme age groups (<40 years and >60 years), where 68.4% and 62.4% were male, respectively. Among females, representation was relatively higher in the middle-aged groups (41–50 years). Marital status was strongly associated with age; 78.4% of individuals aged 51–60 years were currently living with their spouses compared with 63.3% in the 41–50-year age group. Educational level demonstrated an age gradient, with bachelor’s or higher education predominating among the younger age groups (<50 years), whereas high-school education was more frequent in older participants (>60 years). Regarding employment, more than half of the total respondents (76.3%) were not working, either retired, homemakers, or unemployed, while 23.7% were currently employed. Among those aged >60 years, 76.3% were unemployed, compared to 21.1% in the 41–50 age group, indicating an expected decline in employment with age. The distribution of chronic diseases revealed that 343 (79.7%) participants had one chronic disease, while 87 (20.3%) had two or more chronic diseases (multimorbidity). Out of 430 participants, 109 (25.3%) reported good quality of life (QoL), while 321 (74.7%) reported poor QoL. This finding indicates that nearly three out of every four individuals living with chronic diseases in the Assir region perceive their overall QoL as poor (Table 1).

**Table 1:** Distribution of sociodemographic characteristics of the study participants (*n* = 430).

		Age group							
		<40 years		41–50 years		51–60 years		>60 years	
		n	%	n	%	n	%	n	%
Gender	Male	13	68.4%	56	51.4%	72	62.1%	116	62.4%
	Female	6	31.6%	53	48.6%	44	37.9%	70	37.6%
Marital	Currently living with a spouse	12	63.2%	69	63.3%	91	78.4%	129	69.4%
	Other	7	36.8%	40	36.7%	25	21.6%	57	30.6%
Education	High school	0	0.0%	26	23.9%	19	16.4%	31	16.7%
	College/Diploma	6	31.6%	23	21.1%	22	19.0%	40	21.5%
	Bachelor’s degree	13	68.4%	60	55.0%	75	64.7%	115	61.8%
Chronic disease	One	15	78.9%	83	76.1%	93	80.2%	152	81.7%
	Two or more	4	21.1%	26	23.9%	23	19.8%	34	18.3%
Occupation	Not working	14	73.7%	86	78.9%	79	68.1%	142	76.3%
	Currently employed	5	26.3%	23	21.1%	37	31.9%	44	23.7%
	Total	19	100.0%	109	100.0%	116	100.0%	186	100.0%

Gender: 34.2% of males *versus* 12.1% of females reported good QoL (*p* < 0.001). Employment: 47.7% of employed *versus* 17.8% of unemployed participants reported good QoL (*p* < 0.001). Marital status: Married individuals had better QoL (29.6%) compared with those unmarried, separated, or widowed (15.5%) (*p* < 0.002). Education: Participants with higher studies reported the best QoL (48.7%), followed by college/diploma holders (22.0%) and bachelor’s degree holders (19.8%) (*p* < 0.001). Number of chronic diseases: Participants with a single chronic disease reported substantially better QoL (31.2%) than those with two or more chronic diseases (2.3%) (*p* < 0.001). Age group: QoL was lowest in the youngest age group (<40 years, 10.5%) and highest in the 51–60-year group (31.7%), though the difference was not statistically significant (*p* > 0.079).

Gender, marital status, education level, occupation, and number of chronic diseases were significant predictors of QoL. Females, unemployed individuals, and participants with multiple chronic diseases were more likely to experience poor HRQoL across all domains (Table 2).

**Table 2:** Association between sociodemographic characteristics and overall quality of life (*n* = 430).

		Quality of Life						
		Good		Poor		Total		
		n	%	n	%	n	%	*p*-value
Gender	Male	88	34.20%	169	65.80%	257	100%	<0.0001
	Female	21	12.10%	152	87.90%	173	100%	
	Total	109	25.30%	321	74.70%	430	100%	
Working	Currently employed	52	47.70%	57	52.30%	109	100%	<0.0001
	Not working	57	17.80%	264	82.20%	321	100%	
	Total	109	25.30%	321	74.70%	430	100%	
Marital	Currently living with a spouse	89	29.60%	212	70.40%	301	100%	<0.002
	Other (unmarried, separated, widow)	20	15.50%	109	84.50%	129	100%	
	Total	109	25.30%	321	74.70%	430	100%	
Education	Higher study	37	48.70%	39	51.30%	76	100%	<0.0001
	College/Diploma	20	22.00%	71	78.00%	91	100%	
	Bachelor’s degree	52	19.80%	211	80.20%	263	100%	
	Total	109	25.30%	321	74.70%	430	100%	<0.0001
Diseases	One chronic disease	107	31.20%	236	68.80%	343	100%	
	Two or more chronic diseases	2	2.30%	85	97.70%	87	100%	
	Total	109	25.30%	321	74.70%	430	100%	
Age group	<40 yrs	2	10.5%	17	89.5%	19	100%	>0.079
	41–50 yr	21	19.3%	88	80.7%	109	100%	
	51–60 Yr	33	31.7%	71	68.3%	104	100%	
	>60 yrs	53	26.8%	145	73.2%	198	100%	
	Total	109	25.3%	321	74.7%	430	100%	

Multivariable logistic regression analysis revealed multiple independent correlates of good HRQoL (Table 3). Being male, employed, living with spouse, having higher education level, and having one chronic disease were all associated with significantly increased odds of good HRQoL compared to the referent group. Compared with females, males were more likely to report good QoL (aOR = 2.46, 95% CI [1.64–3.68], *p* < 0.001). Good QoL was also more likely among participants who were currently employed *versus* not working (aOR = 1.57, 95% CI [1.31–1.89], *p* < 0.001). Participants who were currently living with a spouse had better QoL compared with participants unmarried, divorced, or widowed (aOR = 1.85, 95% CI [1.21–2.83], *p* < 0.004). Higher level of education was associated with increased odds of good QoL (aOR = 2.39, 95% CI [1.53–3.74], *p* < 0.001). Finally, participants with one chronic disease had substantially higher odds of good QoL compared with participants with multimorbidity (aOR = 45.99, 95% CI [10.04–210.71], *p* < 0.001). The Hosmer–Lemeshow test showed good model fit (*p* = 0.684), and no evidence of multicollinearity was found (VIF < 2.5 for all variables).

**Table 3:** Multivariable logistic regression analysis of factors associated with good health-related quality of life (HRQoL) (*n* = 430).

Variable	Adjusted OR	95% CI	*p*-value
Gender (Male *vs* Female)	2.46	1.64–3.68	<0.001
Working (Working *vs* Not working)	1.57	1.31–1.89	<0.001
Marital (Married *vs* Other)	1.85	1.21–2.83	<0.001
Education	2.39	1.53–3.74	<0.001
Single chronic disease *vs*≥2	45.99	10.04–210.71	<0.001

**Notes.**

Outcome variable coded as Good HRQoL = 1 and Poor HRQoL = 0. Reference categories: Female (gender), not working (employment), other (unmarried/divorced/widowed), Lower education level, and ≥2 chronic diseases. Adjusted odds ratios (Adj OR) were obtained from multivariable logistic regression.

Analysis of Variance (ANOVA) was conducted to evaluate differences in mean scores across the four WHOQOL-BREF domains (Table 4). Participants with single chronic diseases consistently exhibited higher mean scores than those with multimorbidity across all domains. The mean QoL scores across all domains were significantly lower for participants with multiple chronic conditions (*p* < 0.001 for all). This pattern highlights the cumulative burden of multimorbidity on physical capacity, psychological well-being, and social and environmental adaptation.

**Table 4:** Comparison of WHOQOL-BREF domain scores between single and multiple chronic disease groups (*n* = 430).

**Domain**	**Single morbidity** **Mean (SD)**	**Multimorbidity** **Mean (SD)**	***p*-value**
Physical health	13.79 (3.89)	10.71 (4.02)	<0.001
Psychological health	11.04 (4.12)	7.32 (4.08)	<0.001
Social relationships	10.59 (3.98)	8.14 (3.76)	<0.001
Environmental	12.88 (4.32)	9.37 (4.45)	<0.001

**Figure 1:**
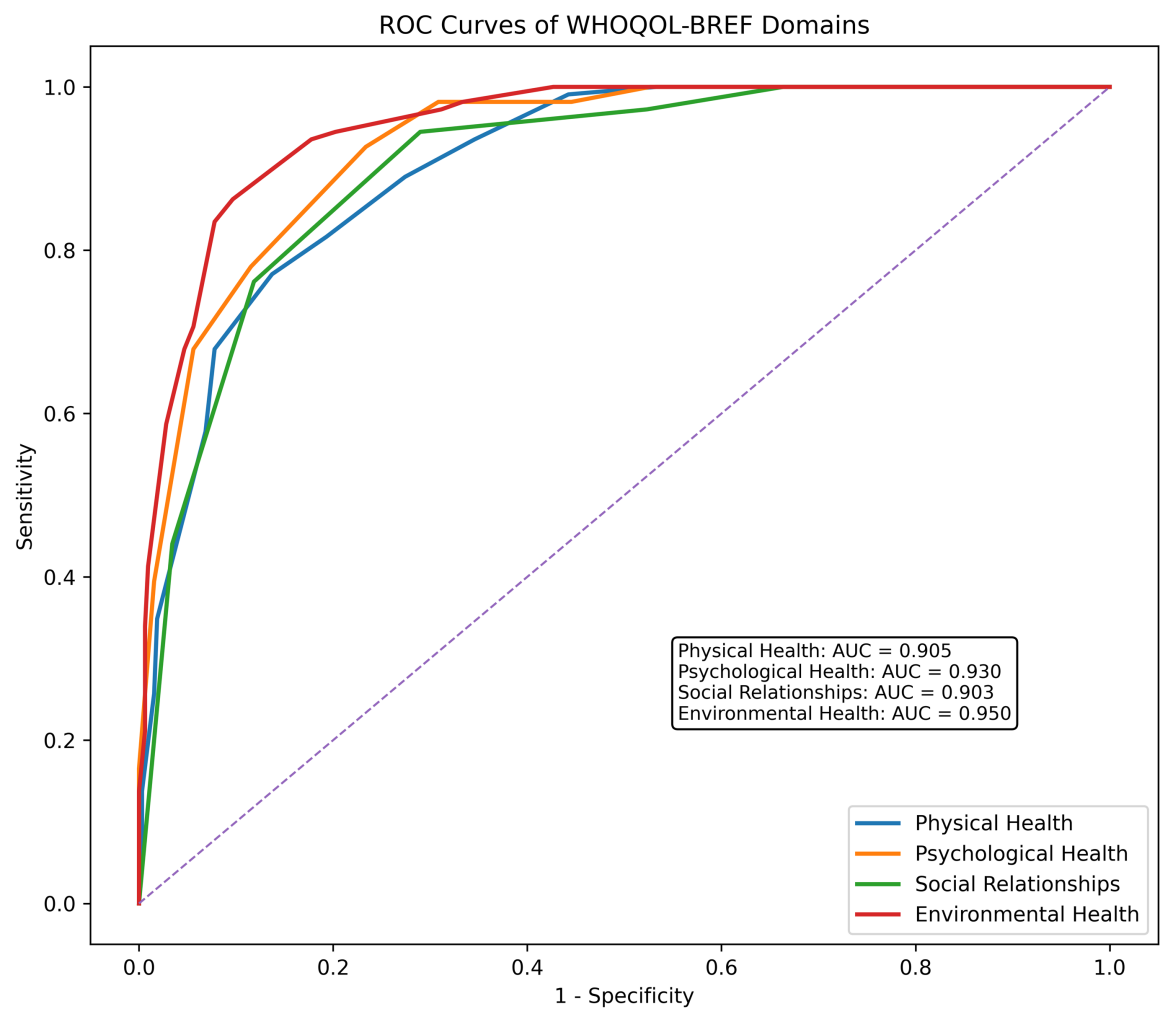
ROC curve of WHOQOL-BREF domines distribution.

Receiver operating characteristic (ROC) analysis revealed that all WHOQOL-BREF domains significantly discriminated between study participants with good and poor HRQoL (Fig. 1). [**State/Outcome variable:** Quality; **Positive state value (event):** “Good”] Area under the ROC curve (AUC) values were highest for the Environmental Health domain (AUC = 0.950), followed by Psychological Health (AUC = 0.930), Physical Health (AUC = 0.905), and Social Relationships domains (AUC = 0.903). Because all domains had excellent AUC values (>0.90), the discriminant validity of the WHOQOL-BREF domains in detecting differences in HRQoL among chronic disease patients was confirmed.

## Discussion

This study comprehensively evaluated the health-Related Quality of Life (HRQoL) among adults with chronic diseases in the Assir region of Saudi Arabia using the validated WHOQOL-BREF instrument. The findings revealed that chronic diseases significantly impair HRQoL across physical, psychological, social, and environmental domains. Furthermore, female gender, unemployment, and multimorbidity were identified as independently associated factors of poorer HRQoL.

These findings are consistent with global and regional evidence showing that chronic diseases contribute to profound physical limitations, psychological distress, and social isolation (Alzarea et al., 2024; Alanazi, 2020; Guallar-Castillón et al., 2005; Ausín, Zamorano & Muñoz, 2020). This is the first study from a predominantly rural Saudi population utilizing the multidomain WHOQOL-BREF instrument, thus filling a critical knowledge gap identified in earlier literature (Alzarea et al., 2024; Samal et al., 2021; Ijoma et al., 2019).

### Gender differences

Male participants were significantly more likely to report good HRQoL than female participants (aOR = 2.46, 95% CI [1.64–3.68], *p* < 0.001), indicating a substantially lower likelihood of good HRQoL among women. This outcome aligns with Guallar-Castillón et al. (2005), who reported poorer QoL among older women. Cultural and social factors in Saudi Arabia may compound these disparities: limited employment opportunities, caregiving responsibilities, and restricted mobility were associated with elevated stress and reduced life satisfaction (Elshamy et al., 2023). Similar gender gaps have also been reported by Farzianpour et al. (2015) and Ausín, Zamorano & Muñoz (2020) in other contexts, confirming that female chronic-disease patients experience greater psychological strain.

### Employment status

Employment emerged as a strong determinant of QoL. Employed participants had 57% higher odds of reporting good HRQoL than those not working (aOR = 1.57, 95% CI [1.31–1.89], *p* < 0.001). Employment enhances financial stability and social participation, key elements for psychological well-being (Alzarea et al., 2024; Elshamy et al., 2023; Alavinia & Burdorf, 2008; Almarabheh et al., 2023; Bratu et al., 2023; Sahoo et al., 2025; Al-Hanawi, 2021). Loss of employment due to chronic illness was associated with reduced income, social withdrawal, and diminished self-esteem, consistent with Alavinia & Burdorf (2008), who observed similar relationships between unemployment and poor health outcomes across Europe.

### Effect of multimorbidity

Participants with a single chronic disease had substantially greater odds of reporting good HRQoL than those with multimorbidity (aOR = 45.99, 95% CI [10.04–210.71], *p* < 0.001), highlighting the considerable adverse impact of multimorbidity on quality of life. Participants with ≥ 2 chronic diseases recorded markedly lower domain scores, particularly in physical and psychological health. This agrees with regional findings by Ijoma et al. (2019) and Alzarea et al. (2024), who documented deteriorating QoL among multimorbid patients. The presence of multiple illnesses was associated with greater treatment complexity, higher medication load, and increased dependency, all of which were linked to diminished perceived well-being (Samal et al., 2021). Our results reinforce the need for integrated chronic-care pathways that simultaneously address physical and mental health aspects.

### Education and marital status

Participants with higher education reported better HRQoL scores than those with lower education levels. Educated individuals often possess greater health literacy and disease-management skills (Gil-Lacruz, Gil-Lacruz & Gracia-Pérez, 2020; Nielsen et al., 2016). However, some evidence indicates that lower education can sometimes correspond to higher perceived QoL, possibly because of lower health expectations or reduced awareness of disease complications (Alzarea et al., 2024; Nielsen et al., 2016). Marital status also influenced HRQoL: married participants demonstrated better QoL than those living alone or widowed, a finding consistent with Farzianpour et al., (2015) and Ausín, Zamorano & Muñoz (2020). Social companionship and spousal support enhance emotional stability and treatment adherence (Elshamy et al., 2023; Alavinia & Burdorf, 2008).

Education was strongly associated with HRQoL in our study. Participants who had higher levels of education enjoyed better QoL. Similar associations between education and QoL have been reported in literature, with Gil-Lacruz, Gil-Lacruz & Gracia-Pérez (2020) and Nielsen et al. (2016) showing how education levels influence patients’ health literacy and ability to self-manage their disease. Nonetheless, some of our respondents who scored poorly in education also reported acceptable QoL scores. It is possible that these individuals have lower expectations of health or are less aware of limitations caused by their disease (Alzarea et al., 2024). With regards to employment status, our unemployed participants (mostly retired due to illness) had significantly lower scores, likely because one’s economic productivity and work are tied to one’s sense of self and psychological well-being, as well as treatment adherence (Alzarea et al., 2024; Alavinia & Burdorf, 2008).

### Domain-specific observations

The physical health domain is moderately affected among participants, especially among multimorbid participants. Similar observations were made by the earlier research by Ausín, Zamorano & Muñoz (2020) that chronic diseases like diabetes, hypertension, and cardiovascular diseases are linked to physical limitations, depending on medication, and increased weakness, all will negatively influence general physical health. Khan et al. (2024) also noted that among Bangladeshi individuals who were suffering from two or more chronic diseases reported lower mean physical health scores compared with a single disease (Khan et al., 2024).

Psychological well-being was severely affected among study participants. The high level of anxiety and depression, of psychological distress, was observed among chronic disease participants. These findings were consistent with previous similar studies by Turner & Kelly (2000) and Vilhena et al. (2014). These chronic diseases can cause emotional exhaustion and diminished self-esteem due to the continuous demands of managing long-term illnesses. Further, the observations advocate that females are more likely to report poorer psychological well-being than their male counterparts. This gender disparity may be due to societal and cultural expectations, particularly in Saudi Arabia, where women may face additional psychosocial stressors related to caregiving roles and limited social engagement, similar to Elshamy et al. (2023) study.

Social relationships and environmental factors are the other domains in which chronic disease experiences challenges. Poorer QoL is reported among unmarried or divorced individuals, where the limited social support is present. These findings are consistence with earlier studies, which highlight that social networks play a critical role in mitigating the psychological and emotional impacts of chronic illnesses (Vilhena et al., 2014). Environmental factors, like access to healthcare services and financial resources, were observed to have a significant influence on HRQoL. Inadequate healthcare access, particularly in rural areas like Assir, was associated with greater health inequality and reduced capacity for effective chronic-disease self-management (Turner & Kelly, 2000; Megari, 2013; National Academies of Sciences, Engineering, and Medicine et al., 2018).

### Policy and practice implications

**1. Integrated care:** Primary-care models should coordinate physical, psychological, and social services for patients with chronic diseases (Akyirem et al., 2022; Samal et al., 2021).

**2. Gender-responsive programs:** Targeted interventions must support women through counseling, empowerment workshops, and improved access to health resources (Elshamy et al., 2023).

**3. Rural health infrastructure:** Enhancing telemedicine, transportation, and outreach services can improve accessibility (Alzarea et al., 2024; Anawade, Sharma & Gahane, 2024).

**4. Employment rehabilitation:** Introducing part-time or flexible-work options for chronic-disease patients can restore purpose and improve QoL (Bratu et al., 2023).

**5. Health literacy:** Community education campaigns should strengthen patients’ understanding of chronic-disease management (Nielsen et al., 2016).

### Limitations and Strengths

**Limitations:** Cross-sectional design precludes causal inferences. Clinic-based sampling may limit generalizability to all rural residents. Disease-severity indicators were not included, possibly confounding outcomes. Self-reported data are subject to recall and social-desirability bias, and psychological comorbidities were not separately analyzed, which might influence HRQoL.

**Strengths:** First multidomain WHOQOL-BREF study conducted in a rural Saudi setting. Large sample size (*n* = 430) provides sufficient statistical power. Validated Arabic WHOQOL-BREF tool with high reliability. Comprehensive regression and ROC analyses ensuring robust predictive validity. Alignment with Saudi Vision 2030 health-sector objectives, enhancing policy relevance.

## Conclusion

This study shows that chronic diseases markedly reduce quality of life among adults in the Assir region, across all four domains: Physical, psychological, social, and environmental, with most participants reporting poor HRQoL. This was especially among individuals with multimorbidity. Male gender, employment status, higher level of education, living with spouse and having one chronic disease were independently related to better HRQoL, while female gender, unemployment, lower educational level, living without spouse and multimorbidity were associated with lower HRQoL. These findings highlight the need for integrated, gender-responsive, and socially supportive chronic-care strategies. Strengthening rural primary care, enhancing health literacy, and improving access to psychosocial and economic support can help improve HRQoL. By addressing social and environmental determinants, health authorities can advance the objectives of Vision 2030 toward equitable well-being for all Saudi citizens.

## Supplemental Information

10.7717/peerj.21592/supp-1 Supplemental Information 1 SPSS Dataset

10.7717/peerj.21592/supp-2 Supplemental Information 2 STROBE Checklist

## Additional Information and Declarations

### Data Availability

The following information was supplied regarding data availability: The data is available in the Supplemental Files.
