# Supplementary material for: Quality of life and its associated factors among chronic disease patients in Assir, Saudi Arabia: a cross-sectional study
Source: PeerJ. 2026 Jul 28;14:e21592. doi: 10.7717/peerj.21592 (PMC13426334; doi:10.7717/peerj.21592)
Supplement: Supplemental Information 2 [file peerj-14-21592-s002.docx]

STROBE Statement—checklist of items that should be included in reports of observational studies

|  | Item No | Recommendation | Remarks | Page No |  |
| --- | --- | --- | --- | --- | --- |
| **Title and abstract** | 1 | (*a*) Indicate the study’s design with a commonly used term in the title or the abstract | Included | Page 1 |  |
|  |  | (*b*) Provide in the abstract an informative and balanced summary of what was done and what was found | Provided | Page 1 Abstract section |  |
| Introduction | | |  |  | |
| Background/rationale | 2 | Explain the scientific background and rationale for the investigation being reported | Yes | Page 2 background section |  |
| Objectives | 3 | State-specific objectives, including any prespecified hypotheses | Yes | Page 3 Subheading objectives |  |
| Methods | | |  |  | |
| Study design | 4 | Present key elements of study design early in the paper | Yes | Page 3 Methodology section Subheading study design |  |
| Setting | 5 | Describe the setting, locations, and relevant dates, including periods of recruitment, exposure, follow-up, and data collection. | Included | Page 3 Methodology section Subheading study design |  |
| Participants | 6 | (*a*) *Cohort study*—Give the eligibility criteria and the sources and methods of selection of participants. Describe methods of follow-up  *Case-control study*—Give the eligibility criteria and the sources and methods of case ascertainment and control selection. Give the rationale for the choice of cases and controls.  *Cross-sectional study*—Give the eligibility criteria and the sources and methods of selection of participants. | *Cross-sectional study has been provided* | Page 3 Methodology section Subheading study population |  |
|  |  | (*b*) *Cohort study*—For matched studies, give matching criteria and number of exposed and unexposed  *Case-control study*—For matched studies, give matching criteria and the number of controls per case |  |  |  |
| Variables | 7 | Clearly define all outcomes, exposures, predictors, potential confounders, and effect modifiers. Give diagnostic criteria, if applicable. | Yes | Page 3 &4 Methodology section Subheading Data collection tool |  |
| Data sources/ measurement | 8* | For each variable of interest, give sources of data and details of methods of assessment (measurement). Describe the comparability of assessment methods if there is more than one group. | *Yes its included* | Page 4 Methodology section Subheading Data analysis |  |
| Bias | 9 | Describe any efforts to address potential sources of bias | Yes | Page 7 Limitations section |  |
| Study size | 10 | Explain how the study size was arrived at | Yes included | Page 3 Methodology section Subheading Sample size |  |
| Quantitative variables | 11 | Explain how quantitative variables were handled in the analyses. If applicable, describe which groupings were chosen and why | Yes | Page 4 Methodology section Subheading Data analysis |  |
| Statistical methods | 12 | (*a*) Describe all statistical methods, including those used to control for confounding | yes | Page 4 Results section |  |
|  |  | (*b*) Describe any methods used to examine subgroups and interactions | yes | Page 5 Results section |  |
|  |  | (*c*) Explain how missing data were addressed | NA | NA |  |
|  |  | (*d*) *Cohort study*—If applicable, explain how loss to follow-up was addressed  *Case-control study*—If applicable, explain how the matching of cases and controls was addressed  *Cross-sectional study*—If applicable, describe analytical methods taking account of the sampling strategy | Yes included | Page 5 Results section |  |
|  |  | (*e*) Describe any sensitivity analyses | Yes described | Tables 1-4 |  |
| Results | | |  |  | |
| Participants | 13* | (a) Report numbers of individuals at each stage of study—eg numbers potentially eligible, examined for eligibility, confirmed eligible, included in the study, completing follow-up, and analyzed | Yes | Page 4 &6 Results section  Tables 1-4 |  |
|  |  | (b) Give reasons for non-participation at each stage | NA | NA |  |
|  |  | (c) Consider the use of a flow diagram | No | Na |  |
| Descriptive data | 14* | (a) Give characteristics of study participants (eg demographic, clinical, social) and information on exposures and potential confounders | Yes | Page 4 &6 Results section  Tables 1 |  |
|  |  | (b) Indicate the number of participants with missing data for each variable of interest | Yes (none) | NA |  |
|  |  | (c) *Cohort study*—Summarise follow-up time (eg, average and total amount) |  |  |  |
| Outcome data | 15* | *Cohort study*—Report numbers of outcome events or summary measures over time |  |  |  |
|  |  | *Case-control study—*Report numbers in each exposure category, or summary measures of exposure |  |  |  |
|  |  | *Cross-sectional study—*Report numbers of outcome events or summary measures | *Yes* | Page 7-8 Conclusions section |  |
| Main results | 16 | (*a*) Give unadjusted estimates and, if applicable, confounder-adjusted estimates and their precision (eg, 95% confidence interval). Make clear which confounders were adjusted for and why they were included | Yes | Page 4 &6 Results section  And Tables 1-4 |  |
|  |  | (*b*) Report category boundaries when continuous variables were categorized | Yes | Page 4 &6 Results section  and Tables 1-4 |  |
|  |  | (*c*) If relevant, consider translating estimates of relative risk into absolute risk for a meaningful time period | Yes | Page 4 &6 Results section  And Tables 1-4 |  |
| Other analyses | 17 | Report other analyses are done—eg analyses of subgroups and interactions, and sensitivity analyses. | Done | Page 4 &6 Results section  And Tables 1-4 |  |
| Discussion | | |  |  | |
| Key results | 18 | Summarise key results with reference to study objectives | Yes | Page 6 &7 Discussion section |  |
| Limitations | 19 | Discuss the limitations of the study, taking into account sources of potential bias or imprecision. Discuss both the direction and magnitude of any potential bias. | Yes | Page 8  Limitation section |  |
| Interpretation | 20 | Give a cautious overall interpretation of results considering objectives, limitations, multiplicity of analyses, results from similar studies, and other relevant evidence. | Yes | Page 7 Conclusion section |  |
| Generalisability | 21 | Discuss the generalisability (external validity) of the study results | Yes | Page 8  Limitation section |  |
| Other information | | |  |  | |
| Funding | 22 | Give the source of funding and the role of the funders for the present study and, if applicable, for the original study on which the present article is based. | Yes | Page 8  Funding section |  |

*Give information separately for cases and controls in case-control studies and, if applicable, for exposed and unexposed groups in cohort and cross-sectional studies.

**Note:** An Explanation and Elaboration article discusses each checklist item and gives methodological background and published examples of transparent reporting. The STROBE checklist is best used in conjunction with this article (freely available on the Web sites of PLoS Medicine at http://www.plosmedicine.org/, Annals of Internal Medicine at http://www.annals.org/, and Epidemiology at http://www.epidem.com/). Information on the STROBE Initiative is available at www.strobe-statement.org.
